# Experimental evaluation of virtual needle insertion framework with enhanced haptic feedback

**DOI:** 10.1007/s11548-025-03420-2

**Published:** 2025-06-24

**Authors:** Mostafa Selim, Lars Eisenburger, Tom Dijkhuis, Martijn Van Dam, Alexander Broersen, Douwe Dresscher, Jouke Dijkstra, Momen Abayazid

**Affiliations:** 1 Robotics and Mechatronics, University of Twente, Enschede, The Netherlands; 2 Department of Surgery, Leiden University Medical Center, Leiden, The Netherlands; 3 Department of Radiology, Leiden University Medical Center, Leiden, The Netherlands; 4 Department of Medical Imaging, Radboud University Medical Center, Nijmegen, The Netherlands

**Keywords:** Needle steering, User study, Haptic feedback, Image-guidance

## Abstract

****Purpose**:**

Haptic feedback could improve the efficiency of needle insertion procedures by providing surgeons with enhanced sensing and guiding capabilities. A framework has been developed to provide physicians with enhanced haptic feedback during CT-guided needle insertion procedures in oncology.

****Methods**:**

The physicians encountered needle-tissue interaction and guidance forces with visual feedback to accurately reach the tumor. The force feedback to users was enhanced by amplifying several parameters in the feedback model, such as tip forces and radial forces. The study evaluated the effect of multiple haptic and visual feedback algorithms on user performance in efficiently inserting the needle. In this experimental pilot study, 12 participants including three interventional radiologists engaged in five experimental scenarios simulating a needle insertion.

****Results**:**

The results showed that enhanced force feedback for tumor perception reduced tumor targeting error and trajectory deviation, compared to natural force feedback. This was also the case when tumor perception and haptic guidance were both enhanced. Additionally, real-time visual feedback and enhanced force feedback for guidance reduced the duration to finish the task significantly. Participants still preferred real-time visual feedback over enhanced haptic feedback cues.

****Conclusions**:**

On average, small tumors (around 3*mm* in diameter) can be successfully targeted with enhanced haptic feedback in the radial and axial directions. Additionally, critical regions, such as veins within the liver, can be avoided more effectively as users maintain the desired trajectory with greater accuracy.

## Introduction

Robotic-assisted surgery is a major advancement in minimally invasive procedures, with increasing adoption expected [1]. In particular, teleoperation enables remote interventions by leveraging wireless networks and robotics [2], allowing expert surgeons to operate from a distance.

Interventional radiology, a growing field within minimally invasive surgery, has shown great potential for robotics integration. This advancement enhances precision, improves safety, and reduces radiation exposure [3]. Moreover, teleoperation could be incorporated into interventional radiology workflows [3]. However, progress remains slow due to high costs, complex equipment, and the absence of haptic feedback [3, 4]. Haptic feedback, which provides force or tactile sensations to inform users about their interactions [5], is widely used in consumer products. Yet, its integration into medical procedures is still uncommon, primarily due to unreliable implementation [6].

While teleoperation has helped surgeons avoid hazardous radiation in CT-guided procedures [7], it eliminates direct physical interaction with the patient [8]. Without haptic feedback, surgeons must rely solely on visual cues to estimate force application, making it difficult to perceive critical sensations, such as tissue texture and instrument collisions [9, 10]. By incorporating haptic feedback, surgeons can better regulate applied forces, reducing the risk of tissue damage caused by excessive force or tension [11]. Although achieving realism and designing effective user studies remain challenging, research into haptic feedback in surgery continues to hold significant promise [12].

Various haptic feedback modalities for needle insertion have been studied extensively. Kinesthetic feedback reliably guides predetermined paths [13, 14], while magnified force feedback helps differentiate soft tissues of varying stiffness [15]. Most haptic feedback systems for needle insertion have been tested with real-time modalities, such as Ultrasound imaging (virtual or real) [13, 15–17]. However, haptic feedback is particularly valuable when real-time visual feedback is limited, as users rely on it for needle control [18], highlighting differences between different forms of force feedback. CT-guided procedures, for instance, require restricted visual feedback to minimize radiation exposure. Prior research has yet to determine whether enhanced force feedback for tumor perception and guidance can reduce interventional radiologists’ dependence on CT scans and improve performance.

In this study, we investigated this research question by building on the work of [19] which developed enhanced haptic and visual feedback for virtual needle insertion procedures. Cross-sectional liver meshes simulated a CT-guided procedure, where haptic feedback enabled physicians to sense needle-tissue interaction forces and provided guidance cues through force feedback to align the needle with the desired trajectory toward the tumor.

### Contribution

This study investigated the impact of enhanced haptic feedback on the performance of novice and experienced medical professionals during needle insertions. The feedback was delivered via a single kinesthetic haptic interface, providing sensing of needle-tissue interaction forces in the axial direction and/or guidance toward the target in the radial direction of the needle. A conservative haptic feedback model was implemented to deliver enhanced force feedback cues for targeting tumors, mitigating excessive forces that could be inconvenient to users and lead to targeting errors. Additionally, the study evaluated the benefits and clinical relevance of the enhanced haptic feedback algorithm in CT-guided needle insertions, incorporating insights from potential end users on its practical utility. Finally, a comparison between experienced and novice participants offers deeper insight into the framework’s applicability for both current and future interventionalists.

## Materials & methods

### Setup & virtual simulator

**Fig. 1:**
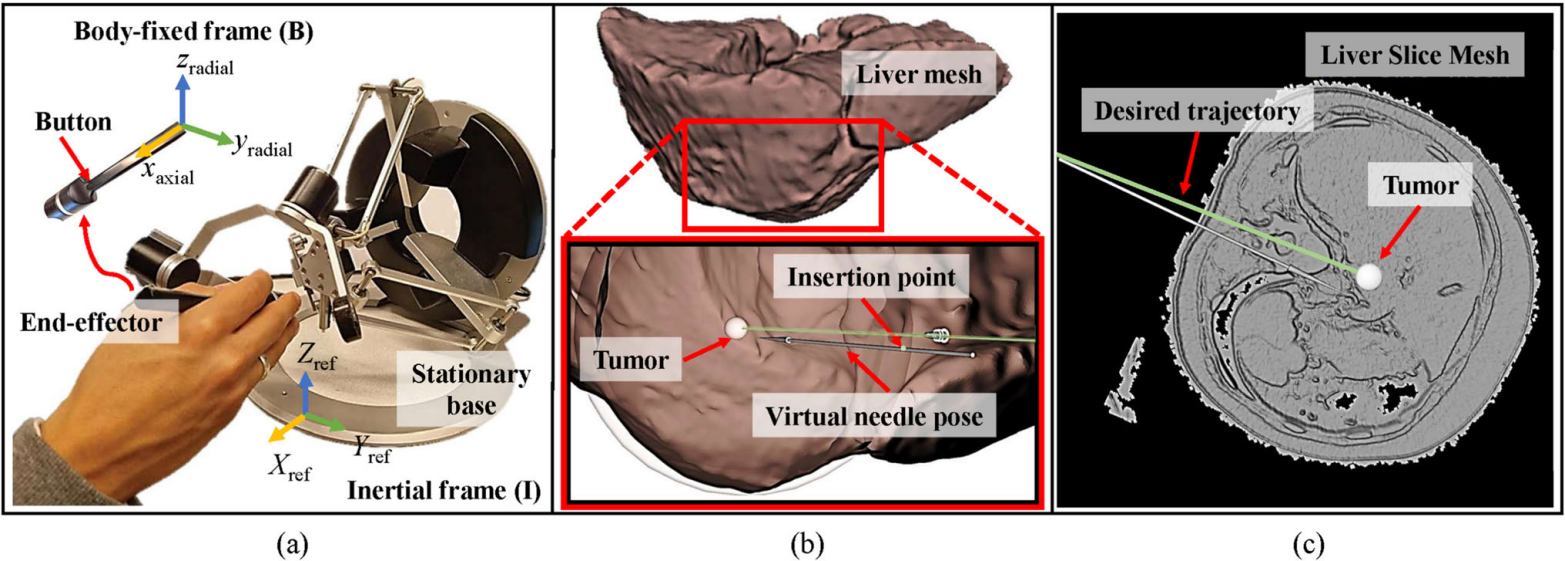
(a) Omega.6 haptic device controlled the virtual needle, (b) a liver mesh exterior view with an arrow imitating the pose of the needle, and (c) a cross-section of a liver mesh showing the interior structure of the liver highlighting the tumor target and the desired trajectory ($\copyright$ 2024, *MDPI*, *sensors*) [19]

We used the Omega.6 haptic device in Fig. 1a (Force dimension, Nyon, Switzerland) to provide 3-degrees-of-freedom (DoF) force feedback while controlling the needle pose. It ran on an Ubuntu 20.04 operating system where the simulation was visualized. The simulation and the haptic feedback algorithm were implemented using ROS (Robot Operating System), with the virtual environment visually represented through ROS visualization (RViz). The liver model consisted of three structures, namely, liver capsule, liver tissue, and tumor. The liver, shown in Fig. 1b, is visible in the simulation, but the actual location of the tumor was blurred during the experiment. For that reason, scans shown in Fig. 1c were requested by participants to visualize the latest pose of the needle relative to the tumor as in CT-guided interventions. The task objective for participants was to position the needle tip as close to the tumor as possible. A trajectory line was visible in the simulation showing the shortest route to the tumor.

### Haptic feedback strategy

**Fig. 2:**
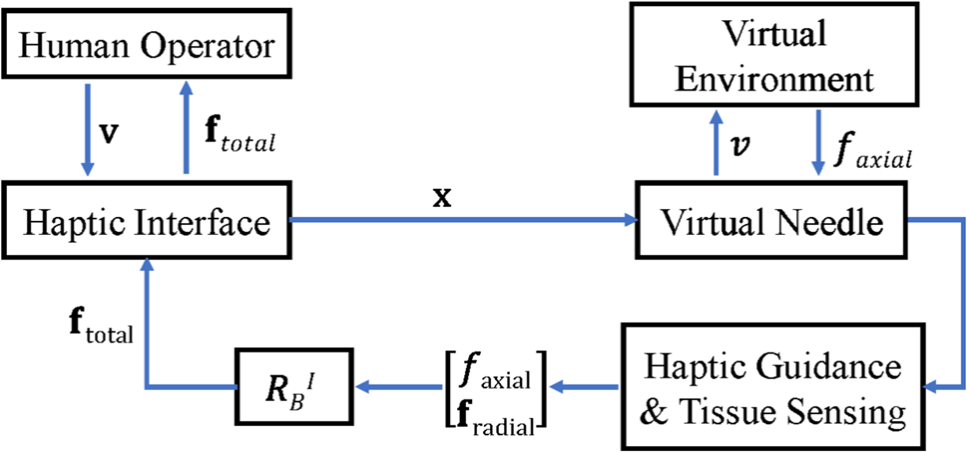
Controller architecture implemented to render forces on the haptic interface

A 3-DoF haptic feedback algorithm was implemented as a position controller in the radial direction of the needle to maintain the desired trajectory and as a force controller in the axial direction to render needle-tissue interaction forces. The orientation of the haptic device handle was used to reorient the needle outside the tissue and then needle orientation was maintained once it penetrated the tissue. Within the tissue, the user could realign the needle with radial motion and insert the needle with axial motion. The controller architecture implemented to render forces to the user is shown in Fig. 2.

#### Radial force feedback

The user was guided through radial forces to maintain smooth insertion along the desired trajectory with minimum tremors. Radial motion was constrained by implementing a Proportional-Derivative (PD) position controller. Equation (1) represents the PD controller,

1 $$\begin{aligned} \textbf{f}_{\text {radial}}=k_{\text {p}}\textbf{d}-k_{\text {d}}\textbf{v}, \end{aligned}$$

where $\textbf{f}_{\text {radial}}\in \mathbb {R}^2$ represents radial forces on the virtual needle in the z–y plane of the body–fixed frame of the needle as shown in Fig. 1. $k_{\text {p}}$ is a constant proportional gain and ${\textbf {d}}\in \mathbb {R}^2$ is the orthogonal vector from the incision point to the virtual needle with some tolerance *t* to move freely in a small cylindrical capsule. *t* enabled the user to realign the needle with the target. The incision point is where the needle tip first penetrates the tissue. $k_{\text {d}}$ is a constant derivative gain and ${\textbf {v}}\in \mathbb {R}^2$ is the radial velocity vector of the needle.

#### Axial force feedback

The proposed force model by [20] was adopted to simulate needle-tissue axial forces of the liver tissues as illustrated in equation (2).

2 $$\begin{aligned} f_{\text {axial}}=f_{\text {stiffness}}+f_{\text {friction}}+f_{\text {cutting}}. \end{aligned}$$

They characterized the axial force models based on needle insertions into a bovine liver with a mounted force sensor on the needle. Three models were presented; a nonlinear spring model for stiffness forces $f_{\text {stiffness}}$, the Karnopp model for friction force $f_{\text {friction}}$, and a constant for cutting forces $f_{\text {cutting}}$ for a certain velocity. In this study, axial force was divided into two parts; needle tip and shaft forces.

(a) Needle Tip Forces: They are the stiffness and cutting forces. To simulate the stiffness–related forces of the liver capsule on the tip, a nonlinear spring model was implemented in Equation (3),

3 $$\begin{aligned} f_{\text {stiffness}}=k_{\text {stiff}}(x_{\text {init}}-x_{\text {cur}})^2+k_{\text {stiff}}(x_{\text {init}}-x_{\text {cur}}), \end{aligned}$$

where $k_{\text {stiff}}$ is the stiffness of the liver capsule, $x_{\text {init}}$ is the initial point of contact of the virtual needle tip with the liver tissue, and $x_{\text {cur}}$ is the current depth of the tip.

Cutting forces were assumed to be a viscous–friction model to account for different velocities of insertion. These forces acted on the needle tip to penetrate through the liver tissues and tumor and are governed by Equation (4),

4 $$\begin{aligned} f_{\text {cutting}}=vk_{\text {cut}}, \end{aligned}$$

where $k_{\text {cut}}$ is the coefficient of cutting forces and *v* is the axial velocity of the needle.

(b) Needle shaft forces: These are the friction forces $f_{\text {friction}}$ that resist needle’s motion through liver tissues and tumor as shown in Equation (5),

5 $$\begin{aligned} f_{\text {friction}} ={\left\{ \begin{array}{ll} f_{\text {static}}, &  \text {if }v<\epsilon \\ f_{\text {dynamic}}+f_{\text {damping}}, &  \text {otherwise} \end{array}\right. } \end{aligned}$$

The friction forces were divided into two stages. The first stage involved constant stiction forces, $f_{\text {static}}$, when the needle moved at an insignificant velocity less than a predefined threshold, $\epsilon$. The second stage involved a position-dependent damping and dynamic friction when the needle moved at a velocity *v* above the set threshold. The dynamic friction $f_{\text {dynamic}}$ is a constant with a magnitude slightly less than that of the stiction forces, while the damping forces are represented in Equation (6),

6 $$\begin{aligned} f_{\text {damping}}=cv(x_{\text {penet}}-x_{\text {cur}}), \end{aligned}$$

where $f_{\text {damping}}$ is the damping force, *c* is the damping coefficient, and $x_{\text {penet}}$ is the point of penetration of the tip into the liver tissue.

**Fig. 3:**
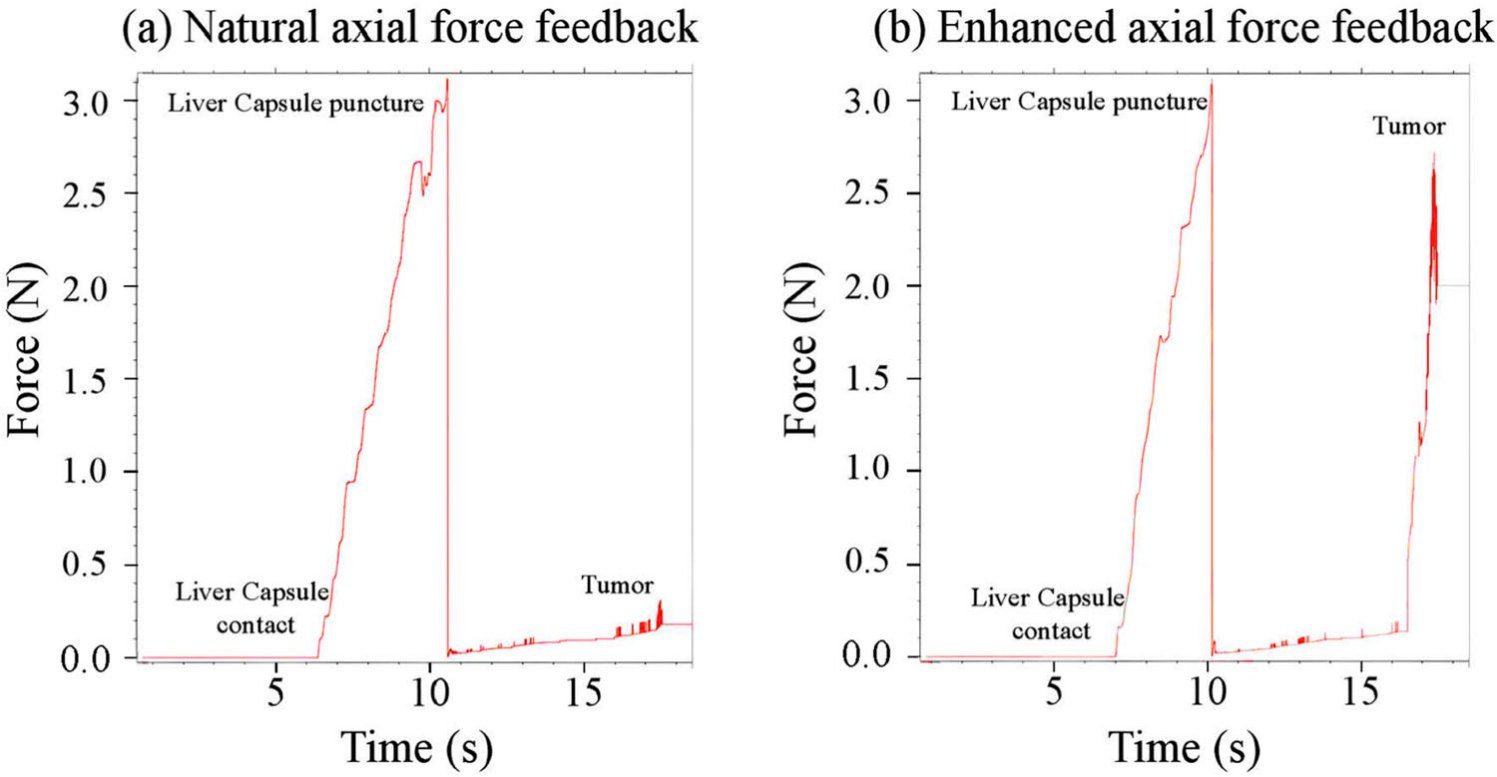
**(a)** Natural axial forces encountered during manual insertion versus **(b)** enhanced axial force feedback to clearly distinguish tumor target

The tumor target was modeled as a liver tissue but with increased stiffness, cutting resistance, and damping forces to simulate a harder tissue structure. The enhanced model parameters were empirically tuned based on user feedback from our previous study [19]. Except for the amplified axial force parameters, all parameters in this study were identical to those used previously. The axial force amplifying parameters were reduced to ensure user comfort and to prevent excessive forces that might push users away from the target, as recommended in our prior study. The enhanced force feedback is shown in Fig. 3 versus the natural axial force feedback in a manual insertion.

#### Modes of operation

Two modes were toggled via a button on the haptic device’s end-effector: one for needle insertion (by holding the button) and another to maintain the needle’s position when the tumor target was reached (by releasing the button). Equation(7) characterizes the PD controller implemented for the second state to keep the handle position stable.

7 $$\begin{aligned} \textbf{f}_{maintain}=k_{\text {p}}\textbf{e}- k_{\text {d}}\textbf{v}, \end{aligned}$$

where $\textbf{f}_{maintain}$ $\in \mathbb {R}^3$ is the force expressed in the inertial frame that kept the end-effector in position and rejected disturbances. The proportional and derivative gains, $k_{\text {p}}$ and $k_{\text {d}}$, are the same magnitude as in Equation (1) and $\textbf{e}\in \mathbb {R}^3$ is the deviation vector of the needle’s tip from the latest position before releasing the button. $\textbf{v}\in \mathbb {R}^3$ is the linear velocity of the needle.

### Experimental design

The experiment evaluated the performance of the participants using various haptic and visual feedback modalities and obtained their feedback as potential operators through a questionnaire. Participants performed five different experimental scenarios.

1. Scenario *BL*, baseline scenario provided compliant radial force feedback after liver capsule penetration, with on-demand cross-sectional liver meshes. Axial forces mimicked natural manual insertion. The simulated tumor had slightly higher stiffness and cutting forces than liver tissue.
2. Scenario *RT*, real-time cross-sectional meshes with compliant radial and natural axial force feedback. This hypothetical scenario contrasts enhanced continuous haptic feedback with real-time visual feedback to assess their effects on user performance
3. Scenario *ES*, enhanced force feedback sensing of the tumor in the axial direction with visual feedback on demand and compliant radial forces. The magnitude of the tumor target parameters were amplified compared to the baseline to enhance tumor perception.
4. Scenario *EG*, enhanced force feedback guidance in radial directions with cross-sectional meshes on demand and natural axial forces. The proportional gain ($k_p$) was amplified in the radial directions relative to its counterpart for the *BL* scenario. Radial force feedback is always active (before and after making contact with the liver capsule).
5. Scenario *ESG*, combined enhanced haptic feedback for sensing and guidance in axial and radial directions, respectively, with visual feedback on demand.

**Fig. 4:**
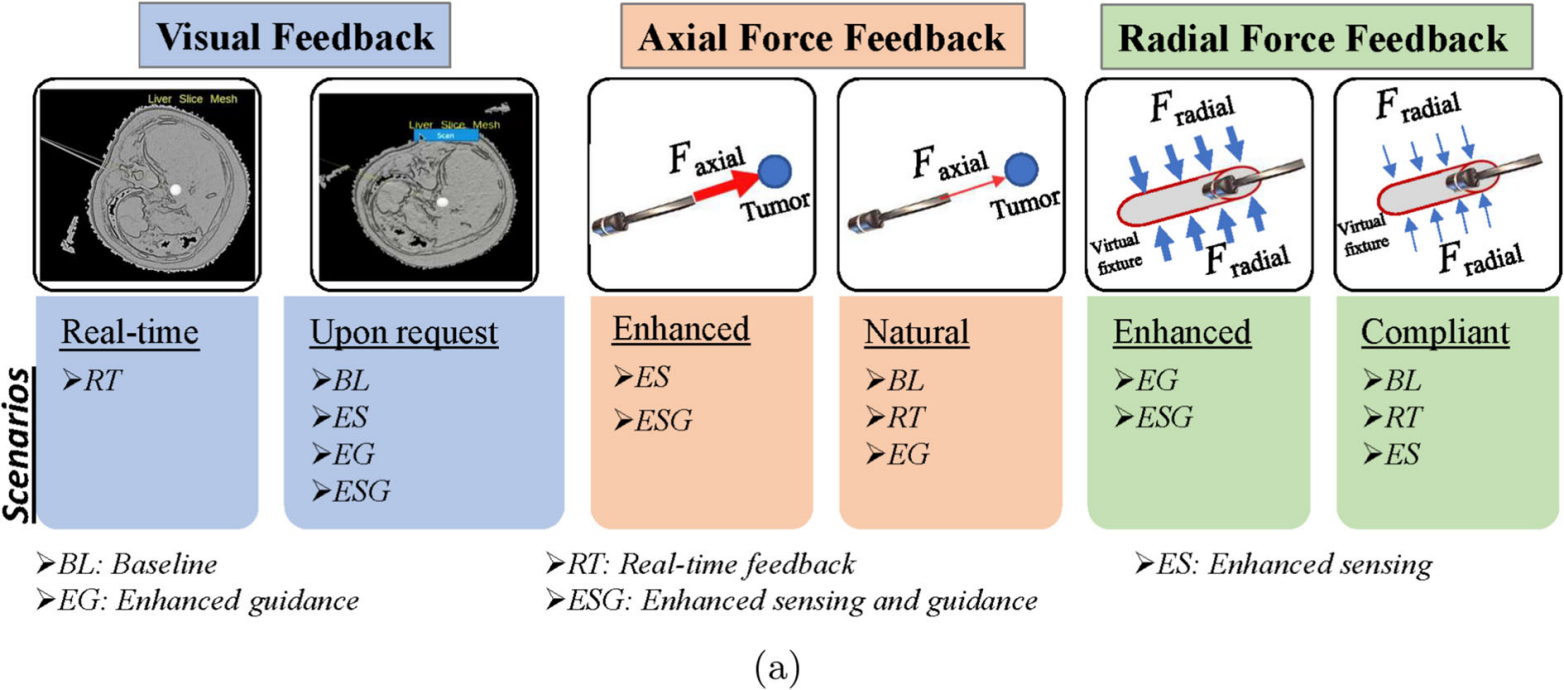
Visual and haptic feedback presented to the user and the experimental scenarios incorporating them

Figure 4 demonstrates the visual and haptic feedback and their integration into the experimental scenarios.

Twelve participants with varying needle insertion expertise took part in the study, including three interventional radiologists, seven junior physicians, and two physician assistants. Four had prior insertion experience. Participants targeted a 2.5*mm* sphere simulating a liver tumor. To reduce learning effects, a tutorial session was provided beforehand. During the experiment, they could request cross-sectional liver meshes and select preferred views to track needle position. Each insertion task was limited to 90 seconds. Minor deviations were corrected with small adjustments, while major ones required needle retraction and reinsertion.

The performance criteria evaluated for this experiment were:

- *The number* of requests for cross-sectional meshes throughout the experiment,
- *Duration* to finish the task with a maximum duration limit of 90 seconds for each trial,
- *Deviation* of the needle tip from the desired trajectory toward the tumor’s centroid. It is the shortest distance between the needle tip and the desired trajectory,
- *Accuracy* of targeting the tumor: final Euclidean displacement error of the needle tip to the target centroid.

### Questionnaire

Participants were surveyed after completing each experimental scenario as well as after finishing all scenarios to gather insights into their experience. A Likert scale from 1 (very dissatisfied) to 5 (very satisfied) was used to evaluate participants satisfaction with various aspects of the experiment. The questionnaire comprised four main topics.

1. Haptic and visual feedback modalities: Participants evaluated the usability of the various visual and haptic feedback modalities after performing each scenario. Answers were collected using two multiple-choice questions and 11 questions using the Likert scale.
2. Overall user experience: Participants were asked eight questions about their general user experience and rated their opinions using the Likert scale.
3. Clinical relevance: Participants assessed the relevance of the haptic feedback algorithm based on four questions with a Likert scale.
4. Improvements and Feedback: To gather participants thoughts on potential improvements and general feedback about the haptic feedback algorithm, three open-ended questions were included.

### Statistical analysis

The collected data were analyzed using IBM SPSS Statistics. A one-way repeated measures ANOVA evaluated the effects of different haptic and visual feedback algorithms on tumor targeting accuracy, trajectory deviation, task completion time, and the number of 2D-mesh requests across five scenarios. Significant outcomes (p< 0.05) were followed by post hoc pairwise comparisons. Descriptive statistics summarized user preferences and mean scores, while qualitative analysis of open-ended responses identified common themes and sentiments, providing additional context to the quantitative findings.

## Results

**Fig. 5:**
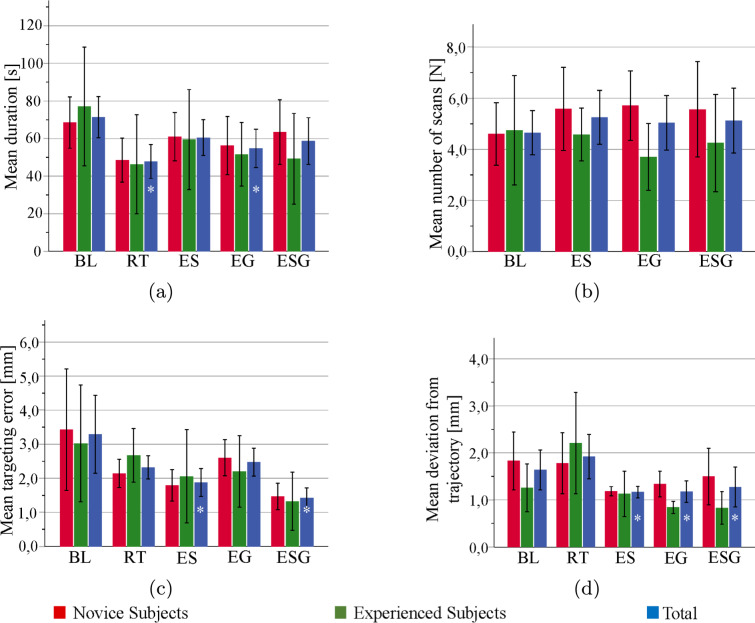
The mean and standard deviation (error bar) over all participants of each scenario for the **(a)** number of scans, **(b)** time needed to finish the task, **(c)** average deviation from the trajectory, and **(d)** tumor targeting error. The scenarios with a $*$ sign showed a significant difference in each metric compared to the *BL* scenario

**Fig. 6:**
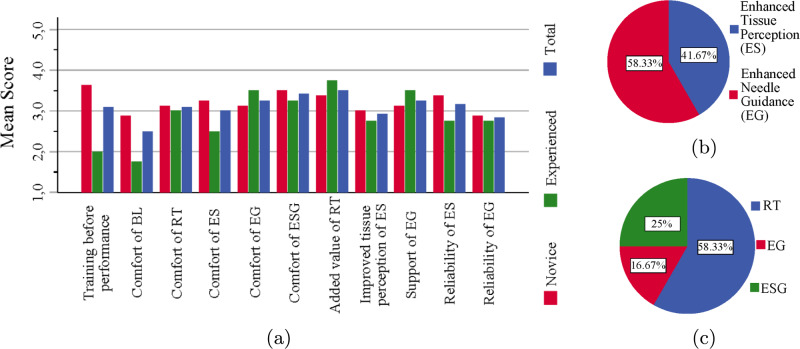
**(a)** Mean score of all participants’ feedback on comfort, perception, and reliability for each experimental scenario. **(b)** Preferred haptic feedback type and **(c)** preferred experimental scenario for all participants

In this section, we present the results from the statistical analysis of user performance shown in Fig. 5 and questionnaire results shown in Fig. 6.

*Duration*: The means of the duration over the five scenarios are shown in Fig. 5a. Participants performed the fastest in Scenario *RT*. The results of the ANOVA test indicated a significant difference in the duration, p=0.023. Relevant post hoc comparisons showed that the insertion task in all scenarios was performed faster than *BL* scenario. Only scenario *RT* and *EG* showed a significant difference relative to the *BL* scenario, p<0.001 and p=0.024, respectively.

*Number of requested liver meshes*: The mean number of requested meshes over four scenarios are shown in Fig. 5b. In Scenarios *ES*, *EG*, and *ESG*, experienced participants requested notably fewer cross-sectional liver meshes than novices. The results of the ANOVA test indicated an insignificant difference in the number of requested 2D-meshes, p=0.665.

*Tumor targeting error*: The means of the targeting error over the five scenarios are shown in Fig. 5c. The targeting error was highest in scenario *BL* and lowest in scenarios *ES* and *ESG*. There are no notable differences between the experienced and novice participants. The results of the ANOVA test indicated a significant difference in the target error, p=0.003. Relevant post hoc comparisons for scenarios *ES* and *ESG* resulted in a significant difference relative to *BL* scenario, p=0.030 and p=0.003, respectively.

*Deviation from the trajectory*: The means of the deviation from the trajectory over the five scenarios are shown in Fig. 5d. The values were highest for scenarios *BL* and *RT* and lowest for scenarios *ES*, *EG*, and *ESG*. The results of the ANOVA test indicated a significant difference in the deviation, p<0.001. Relevant post hoc comparisons showed that scenarios *ES*, *EG*, and *ESG* resulted in a significant difference relative to the baseline, p=0.003, p=0.002, and p=0.032, respectively.

*Haptic feedback modalities*: Fig. 6a shows the mean participant scores after each scenario. Compared to experienced participants, novices found the preliminary explanation and training more beneficial and rated scenario *BL* as more comfortable. In the other scenarios, this was more evened out. Overall, scenario *RT* was the least comfortable and scenario *ESG* was the most comfortable. Haptic feedback for guidance was slightly preferred compared to haptic feedback for enhanced tissue perception. This is illustrated as well in Fig. 6b. Despite lower comfort, most participants favored scenario *RT*, as indicated in Fig. 6c.

**Fig. 7:**
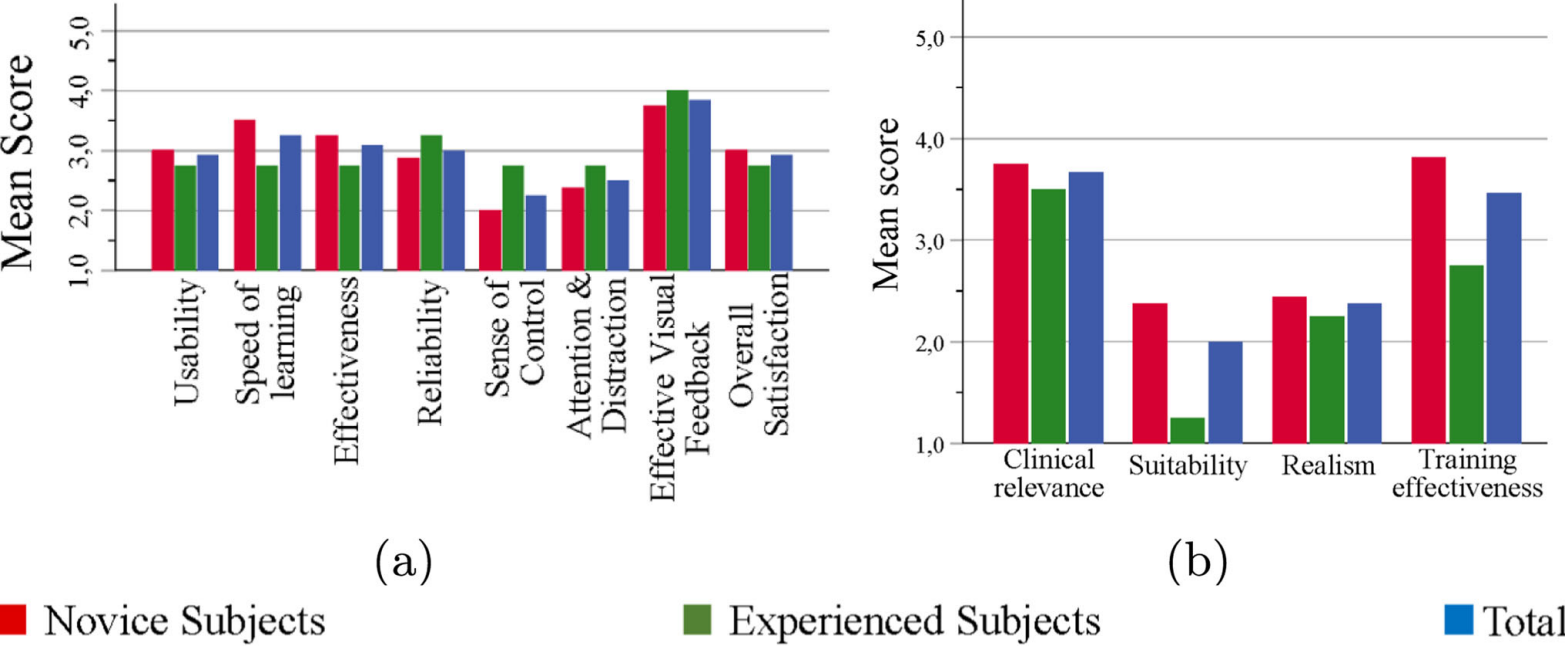
**(a)** Mean score of all participants on the overall user experience and **(b)** clinical applicability

*Overall user experience*: The mean scores of the participants on overall user experience are shown in Fig. 7a. The scores between experienced and novice participants did not differ significantly. The participants benefited greatly from the liver slices when carrying out the experiment. Participants overall satisfaction with the haptic feedback algorithm was neutral.

*Clinical applicability*: The mean scores of the participants on clinical applicability are shown in Fig. 7b. Participants agreed that haptic feedback can be clinically relevant for teleoperated CT-guided needle insertion procedures. However, it was not suitable enough in the tested form to be implemented in the clinic. Furthermore, the experiment was rated as not very realistic. Novice participants felt that a similar approach could be beneficial for medical training, while experienced participants were more reserved about its potential.

## Discussion

The study results suggested that haptic feedback, through enhanced tissue perception and needle guidance, could improve targeting accuracy and trajectory tracking in CT-guided needle insertion simulations. However, real-time visual feedback was preferred over haptic feedback cues. Participants considered the haptic feedback clinically relevant and the enhanced forces appropriate. However, the framework needs further development to enhance the realism of the simulation and imaging. This is because needle deflection and tissue deformation were not included in the simulations, as well as real CT scans. Furthermore, the needle should be aligned with the target by adjusting its orientation and receiving torque feedback.

Participants completed scenario *RT* the fastest, likely due to their familiarity with visual feedback as a primary source of guidance and their increased confidence moving forward toward a continuously visible target. The duration for scenario *RT* also reduced significantly relative to the *BL* scenario. Scenarios *ES* and *ESG* were performed faster than the *BL* scenario but did not reach statistical significance (p=0.057 and p=0.083, respectively). These scenarios were the only ones with haptic feedback for tissue perception. One explanation could be that participants in these scenarios prioritized accurate tumor targeting with enhanced feedback over speeding up to complete the task faster. This was evident in the targeting error results for scenarios *ES* and *ESG*, where the targeting errors were significantly less than *BL* scenario.

The repeated measures ANOVA showed no significant result for the number of requested 2D cross-sectional meshes. Contrary to expectations, enhanced haptic feedback did not reduce participants’ requests for scans. This may be due to the limited consideration given to the utility of the 2D cross-sectional mesh in the *BL* scenario. As the experiment progressed, more meshes were used because they proved useful in earlier scenarios.

Scenarios *ES* and *ESG* showed a significant reduction in targeting error. Haptic feedback for enhanced tissue perception improved tumor targeting accuracy, especially when combined with enhanced haptic guidance. The average deviation from the trajectory was significantly lower in scenarios *EG* and *ESG*. Scenario *ES* showed significantly lower deviation, which was notable given the absence of enhanced guidance.

Questionnaires revealed a preference for real-time visual feedback over enhanced haptic feedback despite the greater accuracy of the later. Participants were generally satisfied with the haptic feedback cues but less pleased with the simulation’s realism.

Finally, we explored remarkable differences between experienced and novice participants. Experienced participants only performed notably worse than novice participants in scenario *RT* regarding the deviation from the trajectory. The reason is likely that experienced participants found the live view more bothersome, despite its additional information. Displaying both external and internal live views may have caused distraction. A unified visual feedback interface integrating both views could help reduce user distraction during the procedure.

Experienced participants found the initial training session and comfort in scenario *BL* significantly less pleasant than novice participants, possibly due to age, as experienced participants were older. Younger individuals typically acquire digital skills faster. Additionally, a notable difference in ’Suitability’ and ’Training effectiveness’ may arise from experienced participants’ familiarity with current practices, influencing their perspective on device and algorithm implementation.

Unlike the previous study [19], participants in this study demonstrated significantly higher accuracy with enhanced haptic feedback, likely due to the moderate amplification of axial forces that maintained needle alignment. However, this did not reduce CT scan usage, contrasting with earlier findings. This may be attributed to medical professionals’ reliance on imaging for guidance and the need for further training to build trust in haptic cues. Ultimately, clinicians prioritize precise tumor targeting over minimizing imaging, as accuracy remains the primary goal.

Finally, the proposed method can be clinically implemented using a robotic manipulator holding the needle with at least 3-DoF to perform insertion and steering. A force sensor mounted on the needle measures needle-tissue interaction forces. Coaxial sensors [21] or Fiber Bragg Grating (FBG) sensors [22] are recommended, as they provide direct measurements of forces at the needle tip, which can be amplified to enhance tumor target perception. To reduce radiation exposure while maintaining visual guidance, real-time imaging can be achieved using ultrasound or synthetic CT [23].

## Conclusion

The study proposed a virtual framework for CT-guided needle insertion into the liver with haptic feedback for feeling needle-tissue interaction forces and guidance toward the target lesion. The study investigated the effect of enhanced haptic feedback on novice and experienced end users and its clinical relevance and benefit. The results suggested that haptic feedback improved insertion efficiency when combining enhanced tumor sensing with needle guidance. On average, small tumors (around 3*mm* in diameter) can be successfully targeted with enhanced haptic feedback in the radial and axial directions. Additionally, critical regions, such as veins within the liver, can be avoided more effectively as users maintain the desired trajectory with greater accuracy. However, it was recommended to improve the realism and suitability of the framework for clinical use. To address the identified limitations, future research should prioritize the integration of advanced models for needle behavior inside the liver, tissue deformation, torque during rotation, and organ movements. These enhancements would boost clinicians confidence in the enhanced haptic feedback algorithm and would encourage them to use it in the clinical workflow. In addition, an extensive user study that includes more experienced clinicians can give more insight to the clinical applicability of such framework.

## Data Availability

Not applicable.
